# Plasminogen Activator Inhibitor-1 4G/5G Polymorphism Presenting as Recurrent Ischemic Stroke: The Microthrombi Shower

**DOI:** 10.7759/cureus.23828

**Published:** 2022-04-04

**Authors:** John Dayco, Taha Ataya, Chad Tidwell, Abdalaziz M Awadelkarim, Rashid Alhusain, Mohammed Ali, Adnan Halboni, John Dawdy, Randy Lieberman

**Affiliations:** 1 Internal Medicine, Wayne State University Detroit Medical Center, Detroit, USA

**Keywords:** magnetic resonance imaging, magnetic resonance angiography (mra), thrombotic coagulopathy, congenital abnormalities, embolic stroke of undetermined source

## Abstract

Certain clinical scenarios should alert a physician to take a deeper look into causative pathological processes. This was evident in the case of a 41-year-old man who presented for recurrent micro thromboembolic strokes, which is atypical for the patient’s age. Our desire to explain the pathological process led to the rare finding of a plasminogen activator inhibitor-1 polymorphism, which has been associated with an increased risk of cerebrovascular thrombosis. A defect in this pathway leads to the inhibition of the tissue plasminogen activator protein. This genetic polymorphism has relatively been unexplored in recent medical literature, and we are hoping that our case may inspire future research that could help potential targets of risk factor stratifications as well as the development of novel pharmacological options.

## Introduction

A key process in the fibrinolytic cascade involves the tissue-type plasminogen activator (tPA)-mediated conversion of plasminogen into plasmin, its active form, which degrades the fibrin within intravascular thrombi. Plasminogen activator inhibitor-1 (PAI-1), which is coded by the SERPINE-1 gene, is an inhibitor of plasminogen activator proteins, such as tPA [1]. An abundance of PAI-1 would lead to a hypercoagulable state through the inhibition of tPA. Conversely, a deficiency of PAI-1 would lead to an increase in tPA activity, leading to a higher propensity for fibrinolysis and bleeding. In this case report, we explore the case of a 41-year-old man, who presented for recurrent micro thromboembolic strokes. An extensive workup was remarkable for a genetic variant that leads to an elevated amount of PAI-1, increasing the patient's propensity for thromboembolic strokes. This case serves as a reminder for physicians to maintain vigilance in identifying etiologies for unusual cases, such as a young adult with recurrent thromboembolic strokes. May the concepts highlighted in this case serve as an inspiration for potential targets of novel risk factor stratifications, as well as targeted pharmacological therapy.

## Case presentation

A 43-year-old man with a past medical history of ischemic cerebrovascular accident (CVA) presented with a chief complaint of ambulation difficulty and bilateral upper and lower extremity numbness that started 24 hours prior to presentation. The patient had an ischemic stroke that occurred two years ago and, at the time, the patient was discharged with medical management of aspirin, atorvastatin, and physical therapy. Since then, the patient returned to the emergency department with similar symptoms. Physical exam revealed an unsteady gait, bilateral leg weakness, which was worse with the left leg, and inability to adduct the right eye on leftward gaze. A computed tomography (CT) head without contrast revealed no signs of an acute hemorrhage. Due to the late presentation, tissue plasminogen activator (TPA) therapy was not administered.

**Figure 1:**
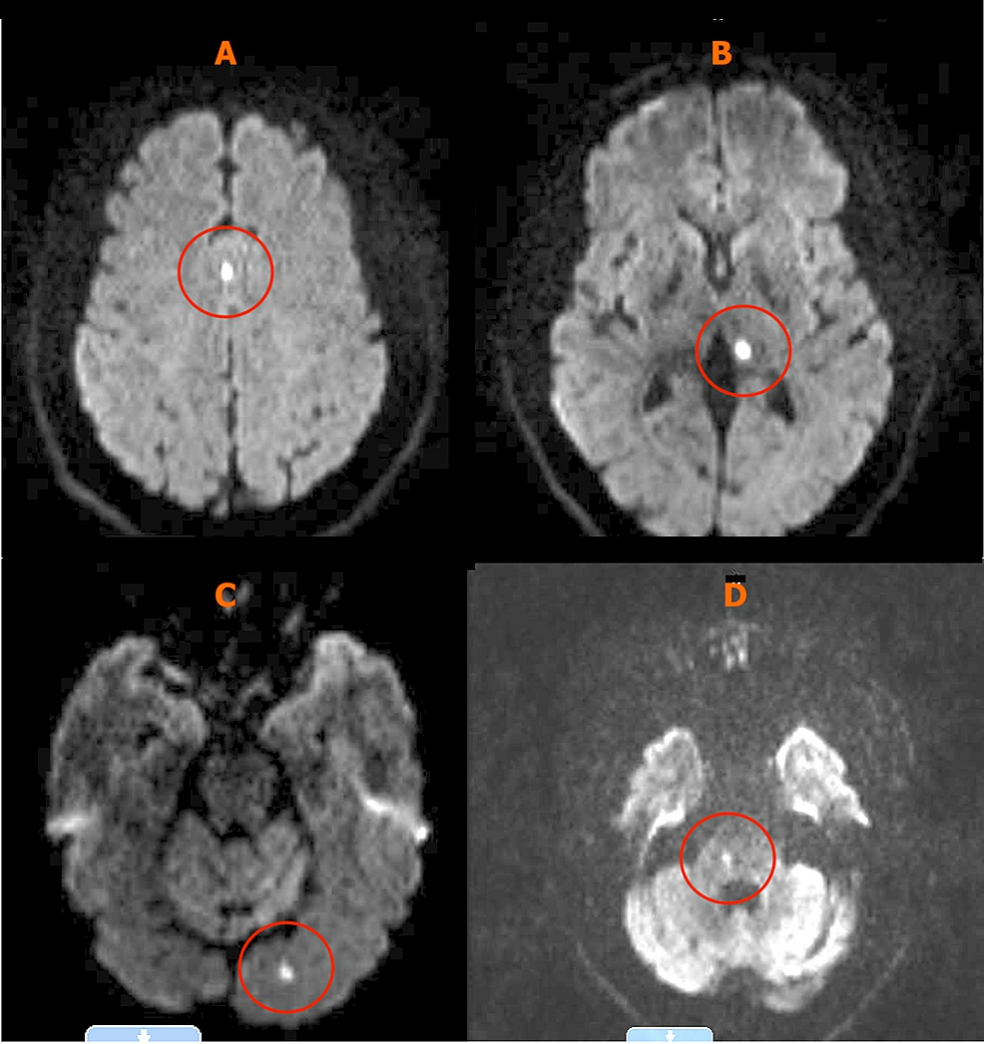
Diffusion-weighted sequence (A-D) demonstrating multiple foci of acute to subacute infarction (circled) within the right cingulate gyrus (A), left dorsomedial thalamus (B), left occipital lobe (C), and right hemi-pons (D).

Further workups, such as transthoracic echocardiogram (TTE) and magnetic resonance angiography (MRA) of the neck were non-remarkable. An electrocardiogram (EKG) revealed normal sinus rhythm. A diffusion-weighted magnetic resonance imaging (MRI) of the brain demonstrated multiple foci of subacute infarction within the right cingulate gyrus, left dorsomedial thalamus, left occipital lobe, and right hemi-pons (Figure 1).

**Figure 2:**
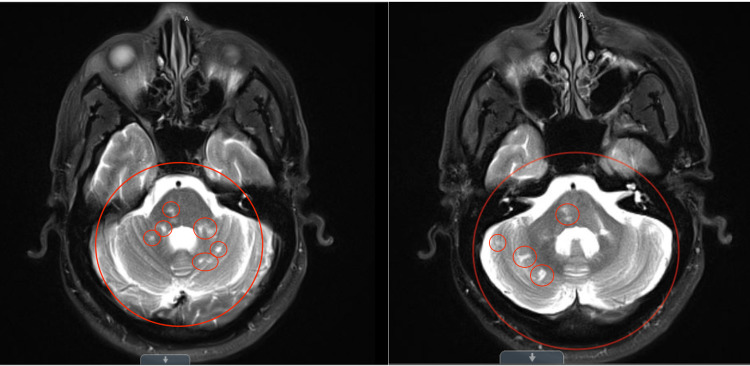
Axial T2-weighted sequence demonstrating remote left middle cerebellar peduncle infarct, and border zone infarcts within the cerebellar hemisphere (circled).

A T2-weighted MRI demonstrated multiple small infarctions located in the left middle cerebellar peduncle and border zones within the cerebellar hemisphere (Figure 2). 

There are evidences of multiple old, micro infarcts in the bilateral cerebellar hemispheres, middle cerebellar peduncles, pons, and thalami. Several studies were performed to explain the etiology of the recurrent stroke. Lipid studies were significant for elevated low-density lipoprotein (LDL) (113), triglyceride (128), and low high-density lipoprotein (HDL) (50). Hemoglobin A1C has been persistently elevated at 9% over the last three years, and due to a history of non-compliance, the patient was only on metformin mono-therapy during presentation. Bilateral carotid duplex imaging was negative for stenosis. There was also no atrial septal defect (ASD) from the TTE bubble study. The hematology service was consulted, which recommended a hematological workup, consisting of prothrombin 20210A, anti-thrombin, protein C, and protein S, anti-cardiolipin, lupus anticoagulant, and anti-beta-2-glycoprotein antibodies, which were all non-remarkable. Further investigations were attempted with a hematologic genetic panel, obtained with a polymerase chain reaction (PCR) using the TaqMan™ assay (Thermo Fisher Scientific, Waltham, Massachusetts, United States). The genetic panel included methylenetetrohydrofolate reductase (MTHFR), Factor II, and Factor V Leiden polymorphisms, which were non-remarkable. However, it did reveal one copy of the 4G variant and one copy of the 5G variant in the SERPINE-1 gene. This genotype is associated with intermediate levels of circulating plasminogen activator inhibitor 1 (PAI-1) protein. Elevated levels of PAI-1 protein has been associated with an increased risk of venous thrombosis. Once this was detected, a likely contributor to the patient's propensity for thrombosis was identified. Once the workup was completed, the patient was then discharged to a rehabilitation facility with aspirin, atorvastatin, and apixaban. The patient will follow up as an outpatient for strict glycemic and lipid control with a particular focus in risk factor modifications.

## Discussion

**Figure 3:**
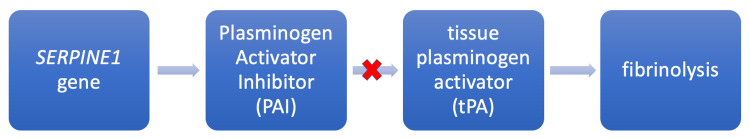
Schematic flow chart illustrating the role of plasminogen activator inhibitor, which inhibits the activity of tissue plasminogen activator, inhibiting fibrinolysis.

PAI-1 is an inhibitor of plasminogen activator proteins, such as tPA. An abundance of PAI-1 would lead to the inhibition of tPA, leading to coagulopathy. A mechanism schematic is demonstrated in Figure 3. 

The PAI-1 4G/5G polymorphism, as coded by the SERPINE-1 gene, had only been recently studied amongst human medicine. However, it does correlate to a higher cerebral venous sinus thrombosis [1]. Most of the available literature discusses PAI-1 deficiency, which is a rare autosomal recessive bleeding disorder that leads to hyperfibrinolysis, commonly seen in spontaneous abortions [2]. Elevated amounts of PAI-1, especially as it relates to multiple strokes in young adults, has not been previously explored.

Our patient, who presented for multiple thromboembolic strokes at the age of 41 years old, warranted further investigation. Prior to the first stroke, the patient only had a past medical history of essential hypertension and type 2 diabetes mellitus. Although the patient presented with two modifiable risk factors for ischemic stroke, adults aged 18-50 years only represent 10-20% of all cases, let alone repeat ischemic stroke [3]. Age is a more significant risk factor in the incidence of stroke at ages 55 years and above. The risk of stroke doubles every consecutive decade over the age 55 [4]. In the case of our patient, his age was unlikely a significant risk factor for stroke. Although this patient did have other co-morbidities that can increase his risk for stroke, it is still an uncommon disease amongst his age group. In addition, the patient suffered a second stroke within two years of the first one. His modifiable risk factors were actively being managed, which would decrease his risk for recurrent ischemic stroke. This repeated presentation in his age group prompted the extensive workup in identifying an etiology.

The hematologic genetic panel was remarkable for one copy of the 4G and one copy of the 5G variant of the PAI-1 gene. Thrombus breakdown requires the transformation of plasminogen to plasmin to degrade the fibrin structure within the thrombus. PAI-1 inhibits this step; thus, a defect in the PAI-1 gene has been linked with coagulopathies and stroke. Although homozygous variants (5G/5G) are not at an increased risk of coagulopathy, heterozygous variants (4G/5G), such as in our patient, are associated with a higher risk for ischemic stroke, especially in the setting of dyslipidemia [5,6]. The 4G/5G polymorphism has also been associated with myocardial infarction in similar patient groups [7]. Another study showed an association between hypertriglyceridemia and elevated serum levels of PAI-1 [8]. The proposed mechanism in this study is the presence of a very-low-density lipoprotein (VLDL) inducible transcription factor (TF) binding to a site in the PAI-1 promoter overlapping the 4G/5G polymorphic site. Therefore, dyslipidemia and hypertriglyceridemia, in the presence of 4G/5G PAI-1 polymorphism, increases the risk for stroke. This patient had elevated triglyceride levels prior to his first stroke, which, in conjunction with his known PAI-1 4G/5G polymorphism, places him at a higher risk. By placing the patient on a high dose statin, significant reductions in his LDL and triglyceride levels were achieved. 

## Conclusions

Since elevated levels of PAI-1 are associated with an increased risk of cerebrovascular disease, this may be a potential target for future risk factor stratifications, as well as targeted pharmacological therapies. Although not widely available, screening options for circulating PAI-1 protein could guide clinicians in establishing appropriate risk factor modification goals. Targeted therapy to decrease the activity of the PAI-1 protein in individuals with increased PAI-1 may mitigate the risk of cerebrovascular accidents and myocardial infarctions. This is a current gap in literature and would need to be addressed in the future. Although the identification of a PAI-1 polymorphism did not change our patient's management consisting of aspirin, statin, and anticoagulation, the identification of this polymorphism led to an emphasis on close outpatient monitoring, tight risk factor modification, and increased attention on preventative measures. 
